# Integrated Analysis of mRNA and miRNA Associated with Reproduction in Female and Male Gonads in Abalone (*Haliotis discus hannai*)

**DOI:** 10.3390/ijms26073235

**Published:** 2025-03-31

**Authors:** Jianfang Huang, Mingcan Zhou, Zhenghan She, Jianming Chen, Caihuan Ke

**Affiliations:** 1 Fujian Key Laboratory on Conservation and Sustainable Utilization of Marine Biodiversity, Fuzhou Institute of Oceanography, College of Geography and Oceanography, Minjiang University, Fuzhou 350108, China; jianfhuang@mju.edu.cn (J.H.); 19835557185@163.com (Z.S.); 2 College of Ocean and Earth Sciences, Xiamen University, Xiamen 361102, China; 22320200155965@stu.xmu.edu.cn

**Keywords:** abalone, aquaculture, transcriptome, gonadal development, reproductive, shellfish

## Abstract

Reproduction and breeding are crucial to maintaining abalone aquaculture. Understanding the molecular underpinnings of sexual maturation is essential for advancing knowledge in reproductive biology. However, the molecular mechanisms of gonadal development in abalones remain poorly understood, particularly in microRNA (miRNA)-mediated regulation. Thus, this study conducted a comprehensive transcriptomic analysis of abalone *Haliotis discus hannai* (*H. discus hannai*) to identify genes and miRNAs associated with ovarian and testicular discovery. This study identified 685 differentially expressed (DE) genes between the *H. discus hannai* ovary (DD_ovary) and testis (DD_testis) groups, comprising 479 upregulated and 206 downregulated genes in the DD_ovary. Moreover, 137 miRNAs, including 83 novel and 54 known miRNAs, were detected, with 30 upregulated and 27 downregulated in the DD_ovary compared to the DD_testis. Bioinformatics analysis revealed that these miRNAs regulate key processes such as carbohydrate metabolic processes, kinase and hydrolase activity, and starch and sucrose metabolism, all potentially associated with reproductive traits. Further, key mRNA candidates, including *Vitelline envelope sperm lysin receptor* (*Verl*) and *Testis-specific serine/threonine-protein kinase* (*Tssk*) 1, and miRNAs such as novel_90 and novel_120, were identified as components of a functional miRNA-mRNA network associated with sexual maturity and sex determination. These key genes were verified using qRT-PCR and fluorescence in situ hybridization (FISH). These transcriptomic and miRNA datasets provide valuable resources for understanding abalone reproductive biology and may support molecular breeding strategies.

## 1. Introduction

Gonadal development is a crucial process of an organism’s life cycle and serves as the basis for reproductive function. In the shellfish aquaculture industry, stable and sustainable growth relies heavily on a consistent supply of high-quality and abundant seedlings [1], necessitating an in-depth understanding of the mechanisms regulating gonadal development. This process is affected by both endogenous and exogenous factors, with endogenous factors including mRNAs and microRNAs (miRNAs) [2,3]. These miRNAs, small non-coding RNAs (18 to 22 nt), control post-transcriptional gene expression via modulation of the 3′untranslated regions (UTRs) of transcripts [4,5]. They play critical roles in development [6], sex identification, differentiation [7], and apoptosis [8]. For instance, miR-125c has been shown to suppress the maturation of immature females by repressing or degradation of vitellogenins and yolk protein expression [9]. However, despite these advances, the role of miRNAs in regulating shellfish reproduction remains poorly understood and requires further research.

Abalones are marine gastropods [10], holding significant economic value in Europe, the United States, and across Asia. In China, abalone constitutes a major mariculture species, with annual production steadily increasing, contributing substantial social and economic benefits. Among these, abalone *Haliotis discus hannai* (*H. discus hannai*) is the dominant species cultivated in Chinese aquaculture [11]. According to the “2023 China Fishery Statistical Yearbook”, the national aquaculture yield of abalone reached an impressive 228,190 tons [12]. Considering its economic and social importance, understanding the regulatory mechanisms of sexual maturation and sex determination in abalone is vital for advancing aquaculture practices. Currently, research on reproductive biology in abalone, including gonadal development, maturation, and gametogenesis, is limited, with the genetic and molecular basis of sexual differentiation and sex-dependent traits remaining poorly characterized. Therefore, more understanding in these areas is essential for optimizing breeding strategies and improving production efficiency.

Recent advancements in modern molecular technologies, particularly high-throughput sequencing (HTS) and bioinformatics, have provided a transformative platform for studying miRNAs. These technologies can identify multiple mature miRNAs and precursor small RNAs, deepening the understanding of gene expression regulation. Moreover, HTS has become a widely adopted approach for exploring gene expression and regulatory mechanisms in various aquaculture species [13,14,15], including studies on reproduction and sex-related genes through RNA-seq technology [16,17,18,19]. However, there remains a significant gap in the genetic information of mRNAs and miRNAs associated with sexual maturation and determination in abalone. In this study, HTS was employed for screening of ovarian and testicular differentially expressed genes (DEGs) as well as miRNAs (DEMs) of *H. discus hannai*. A comprehensive analysis was conducted to integrate these DEGs and DEMs, elucidating their expression profiles and uncovering miRNA–target gene networks associated with sexual maturity and sex determination. These findings offer key insights into the molecular regulation of reproductive traits in *H. discus hannai* and contribute valuable knowledge to an understanding of abalone reproductive biology.

## 2. Results

### 2.1. Overview of RNA-Seq Data

Using the gonads samples of the six *H. discus hannai*, we produced 268,041,282 raw RNA-seq reads. Table 1 displays the RNA quality results. Approximately 260,167,186 clean reads remained after removing reduced-quality, adaptor, and poly-N sequences. We were able to map between 70.86% and 82.94% of the clean reads in each library to the *H. discus hannai* reference genome. Among them, an average of 59.32%, 7.07%, and 33.6% of the mapped reads aligned with exons, introns, and intergenics, respectively.

Furthermore, HTS yielded 11.71 million raw reads, ranging from 11.15 to 12.60 million reads (Table 2). After quality control measures, including the removal of reads with N% > 10%, reduced-quality reads, adaptor sequences, and poly A/T/G/C reads, 11.41 million clean reads (97.43% of the total) were retained for analysis. Among these, 5.02 million sRNAs were annotated, with 89.64% successfully mapped to the *H. discus hannai* reference genome. Moreover, 22 nt sRNAs were the most abundant, underscoring the reliability of our small RNA sequencing methodology. To screen for both reported and new miRNAs in *H. discus hannai,* the small RNA sequences were aligned against mature *Haliotis rufescens* miRNAs from the miRBase database. This analysis identified 54 previously reported and 83 new miRNAs (Table S1).

The raw reads produced in this study were deposited in the NCBI database Sequence Read Archive under the accession number PRJNA1243016.

### 2.2. DEGs and Functional Enrichment Analyses

**Figure 1:**
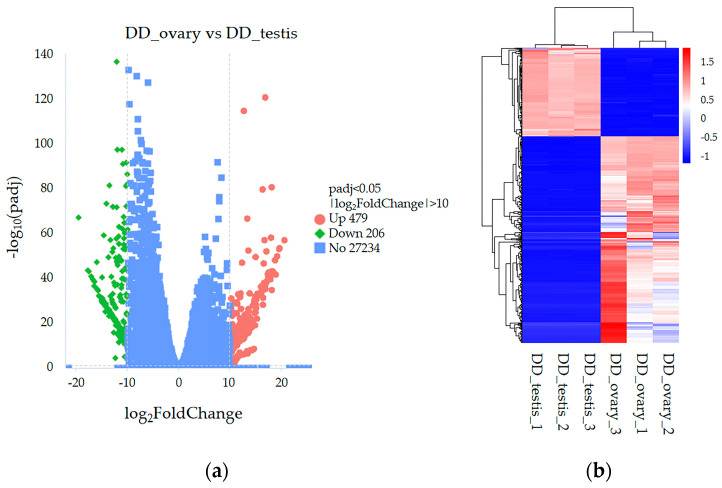
Volcano plots and heatmaps of the mRNA expression of differentially expressed genes (DEGs) in the abalone DD_ovary_group versus the DD_testis_group (*p* < 0.05). (**a**) Volcano plots of DEG expression. Red and green dots indicate up- and downregulated transcripts, respectively. (**b**) Hierarchical clustering of DEGs. Red rectangles represent upregulated mRNAs; blue rectangles represent downregulated mRNAs.

Following annotation, 19,499 ensemble genes were identified in all samples (Table S2). Overall, 685 DEGs were found and expressed in the DD_ovary_group compared with the DD_testis_group (Table S3). Among them, 479 genes were upregulated, and 206 genes were downregulated. Among these upregulated DEGs, the top 50 genes (Table S4) included *Verl*, *Brain tumor protein (brat), Papilin (Ppn), Protein lev-9 (lev-9), Galactoside alpha-(1,2)-fucosyltransferase 2 (Fut2), Tubulin alpha-1A chain (Tuba1A), G2/mitotic-specific cyclin-A (Fragment), Fatty acid synthase (Fasn), Indolethylamine N-methyltransferase (Inmt), Thyroid peroxidase (Tpo), Protocadherin Fat 3 (hFat3), Peroxidasin, Complement Clr-like EGF-like, Lectin C-type domain, Integrase core domain, Zona pellucida-like domain (Zp-like)*, and so on. Among these downregulated DEGs, the top 50 genes (Table S4) included *Tubulin alpha-2/alpha-4 chain*, *Transcription factor SOX-30* (*SOX30*), *Tssk*, *Testis, prostate* and *placenta-expressed protein (Tepp)*, *Kelch-like protein*, *Heat shock factor protein 1* (*HSF1*), *Egg lysin* (*Sperm-lysin*), and so on. The current volcano plots and heatmaps indicated significant differences in mRNA expression (*p* < 0.05) between the groups (Figure 1).

**Figure 2:**
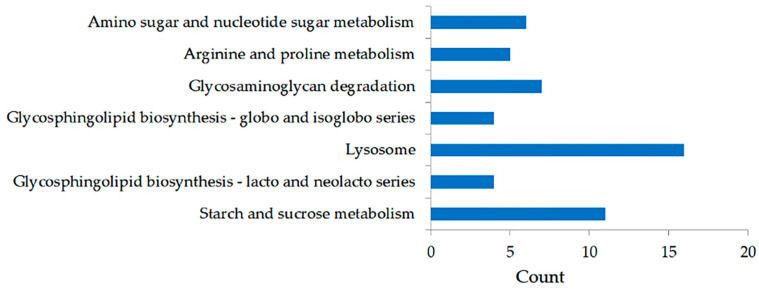
Analysis of significant KEGG pathways for the DEGs.

Both GO and KEGG network enrichment assessments showed the functional significance of the 685 DEGs. These DEGs were considerably associated with 46 GO BPs, 16 GO MFs, and 1 GO CC (Table S5). The BPs included pathways such as carbohydrate metabolic process, protein phosphorylation, phosphorylation, DNA integration, cell–matrix adhesion, etc. Certain DEGs were classified under the CC category, specifically the extracellular region, while others were related to key MFs, including kinase activity, hydrolase activity acting on glycosyl bonds, protein kinase activity, etc. The KEGG pathway analysis identified seven enriched pathways (*p* < 0.05), which included starch and sucrose metabolism, glycosphingolipid biosynthesis (lacto and neolacto series), lysosome, glycosphingolipid biosynthesis (globo and isoglobo series), glycosaminoglycan degradation, arginine and proline metabolism, as well as amino sugar and nucleotide sugar metabolism (Figure 2).

### 2.3. DEMs and Potential miRNA–mRNA Interaction Networks

**Figure 3:**
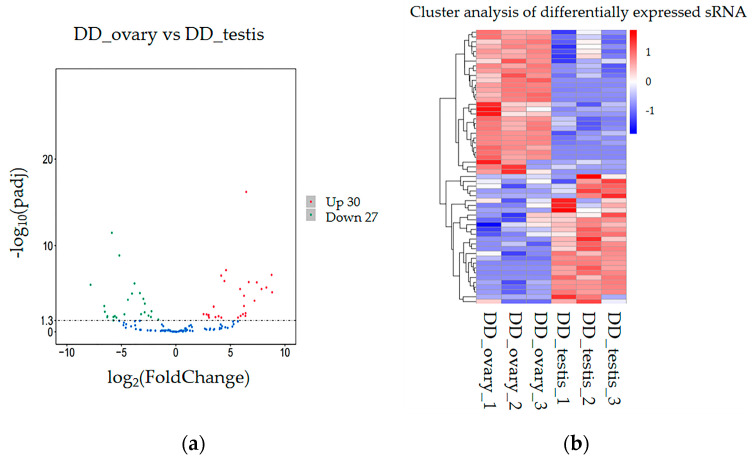
Volcano plots and heatmaps of DE miRNAs (DEMs) in both groups of abalones (*p* < 0.05). (**a**) Volcano plots of DEMs. Red dots depict upregulated miRNAs, while green dots mark downregulated miRNAs. (**b**) Hierarchical clustering of DEMs. Red rectangles depict upregulated miRNAs; blue rectangles show downregulated miRNAs.

Approximately 57 miRNAs were examined as significantly DEMs between the two abalone groups (Table S6). Of these, 30 miRNAs were upregulated and 27 miRNAs were downregulated. The differential expression of these miRNAs was further validated through volcano plots and heatmap analyses, which highlighted significant expression difference (*p* < 0.05) between both groups (Figure 3).

**Figure 4:**
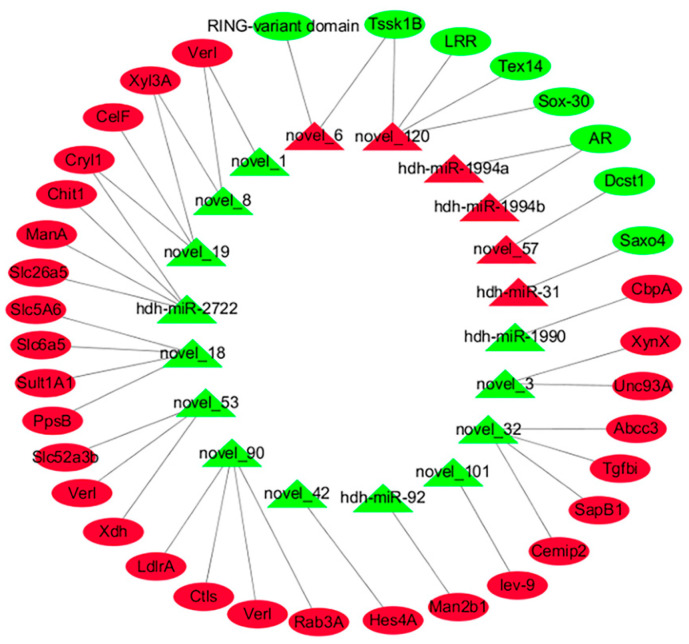
Interaction network of DEMs and selected target DEGs. Green ovals: downregulated target DEGs; red ovals: upregulated target DEGs; green triangles: downregulated DEMs; red triangles: upregulated DEMs.

**Figure 5:**
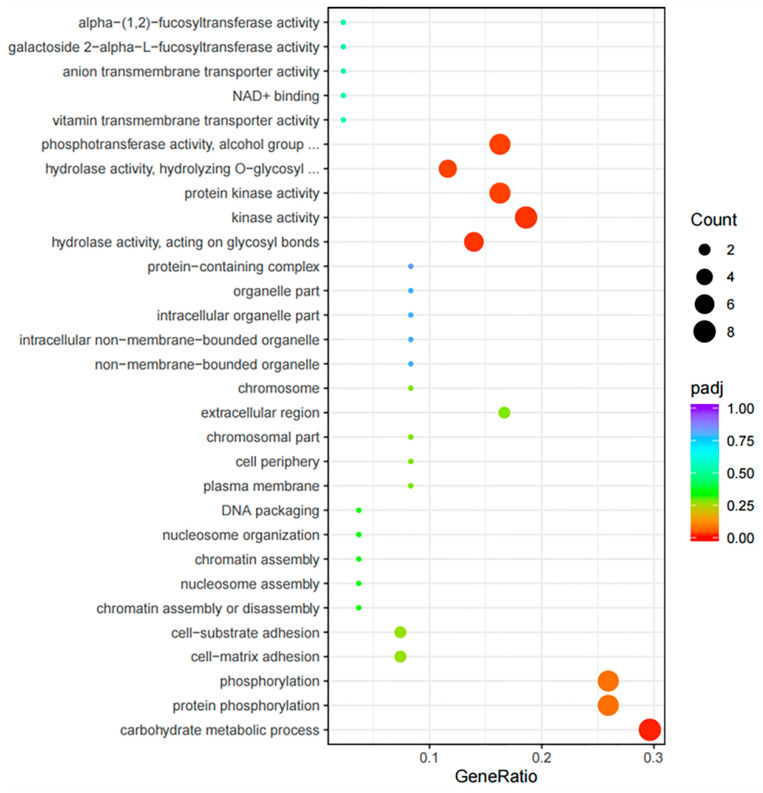
Gene ontology distribution of the target DEGs for DEMs.

To clarify the MFs of the DEMs in both groups, the target genes of 57 DEMs were analyzed. The analysis identified 13 significantly DE target genes (target-DEGs) associated with 7 upregulated miRNAs and 49 target-DEGs related to 16 downregulated miRNAs (Table S7). Interestingly, certain DE-miRNAs were found to target genes related to reproductive processes. For instance, *Verl, Inactive cell surface hyaluronidase CEMIP2* (*Cemip2*), and *Tssk1B* were targeted by novel_90, novel_32, and novel_120, respectively, suggesting that these miRNAs may affect reproductive regulation by modulating these genes (Table 3). Figure 4 illustrates a complex interaction network involving the DE-miRNAs and target-DEGs, highlighting their potential significances in *H. discus hannai* reproductive biology. Moreover, the GO distribution of the predicted target DEGs is depicted in Figure 5. These assessments showed multiple BPs between both groups. Some targets were categorized as BPs, including carbohydrate metabolic process, protein phosphorylation, cell–matrix adhesion, and cell–substrate adhesion. All other targets were related to important MFs, including hydrolase activity acting on glycosyl bonds, kinase activity, protein kinase activity, hydrolase activity hydrolyzing O-glycosyl compounds, and phosphotransferase activity alcohol group as acceptor. A KEGG pathway analysis showed starch and sucrose metabolism significant pathways (*p* < 0.05).

### 2.4. Verification Analysis of Identified DE-mRNA and DE-miRNA

**Figure 6:**
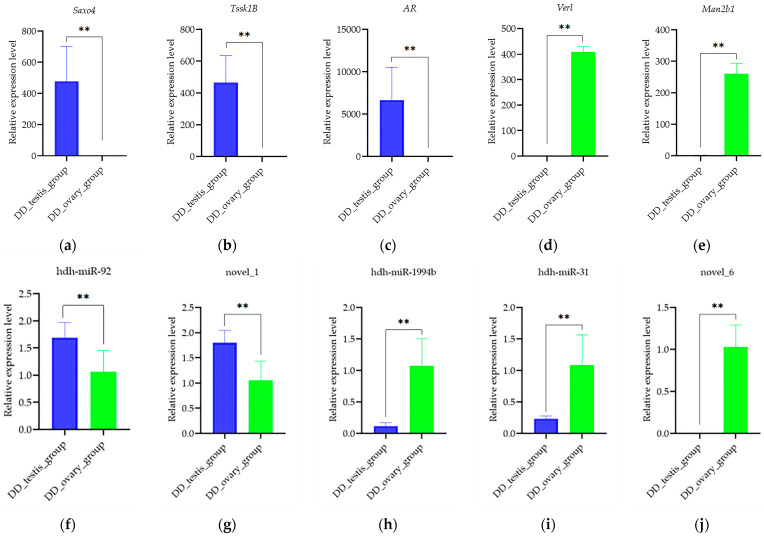
Expression of DEMs and DEGs quantified with qRT-PCR. Data are shown as mean ± SD (*n* = 3). **, *p* < 0.01.

The expression levels of the DEGs (*Saxo4, Tssk1B, AR, Verl,* and *Man2b1*) and DEMs (hdh-miR-92, novel_1, hdh-miR-1994b, hdh-miR-31, and novel_6) were verified using qRT-PCR. The expression patterns of these DEGs and DEMs were consistent with the results of the RNA sequencing (Figure 6), suggesting high reliability of the RNA sequencing analysis.

### 2.5. Localization of Verl and Tssk1B in the Gonad of H. discus hannai

**Figure 7:**
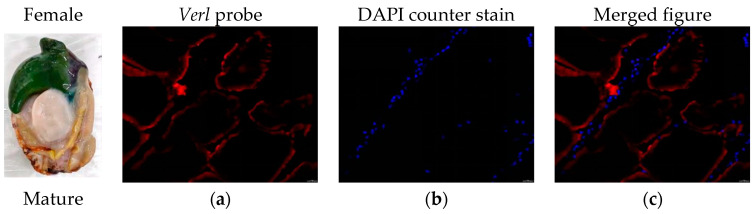

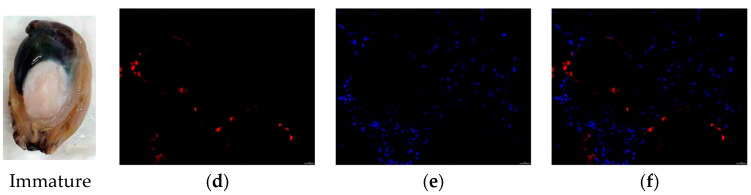
Confocal laser scanning microscopic observation after FISH of *Verl* mRNA in the female gonad tissue of *H. discus hannai*. (**a**,**d**) Fluorescent images of *Verl* antisense probe (red fluorescent). (**b**,**e**) Fluorescent images of DAPI counterstain (blue fluorescent). (**c**,**f**) Fluorescent images of Merged. Scale bars: 50 μm.

**Figure 8:**
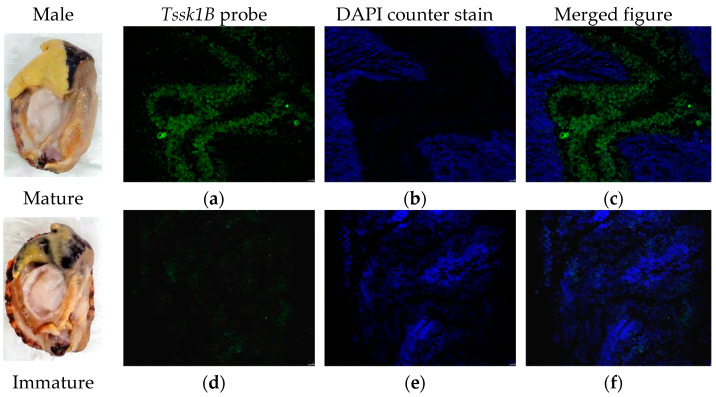
Confocal laser scanning microscopic observation after FISH of *Tssk1B* mRNA in the male gonad tissue of *H. discus hannai*. (**a**,**d**) Fluorescent images of *Tssk1B* antisense probe (green fluorescent). (**b**,**e**) Fluorescent images of DAPI counterstain (blue fluorescent). (**c**,**f**) Fluorescent images of Merged. Scale bars: 50 μm.

FISH was performed to detect the expression of *Verl* and *Tssk1B* mRNAs in the gonadal tissues of *H. discus hannai*. Fluorescence micrographs revealed that the *Verl* antisense probe produced strong positive signals (red; Figure 7a,c) in the gonads of mature females, while only weak positive signals were observed in immature female gonads (Figure 7d,f). Similarly, the *Tssk1B* antisense probe displayed positive signals (green; Figure 8a,c) in the gonads of mature males, but weak signals were detected in the gonads of immature males (Figure 8d,f). Blue fluorescence (DAPI-stained) indicating counterstaining of the positive signal of *Verl* (Figure 7b,e) and *Tssk1B* (Figure 8b,e).

## 3. Discussion

Reproduction is a biological process essential for the survival and propagation of all living organisms. It ensures the continuity of species across successive generations by promoting gamete production and offspring development. Understanding the mechanisms and regulatory pathways involved in reproductive processes is pivotal for advancing animal breeding practices. Enhancing reproductive efficiency in both genders is a crucial strategy in the production process, as it minimizes the number of animals required for breeding, thereby decreasing production costs and optimizing resource consumption. The stable and sustainable growth of the shellfish aquaculture industry relies heavily on a consistent supply of high-quality and abundant seedlings [1]. A comprehensive examination of shellfish reproductive traits is essential to ensure the consistent advancement of seedling production. Currently, several genes and proteins associated with the gonads and reproduction of abalone have been identified using HTS techniques. For example, Mendoza-Porras et al. [20,21] identified genes and proteins associated with sexual maturity, spawning, and reproduction in *H.laevigata*. Moreover, there are also some reports about the genes related to sexual maturity, sex determination, and reproduction in *H. discus hannai* and *H. diversicolor* [22,23,24,25,26]. MicroRNAs (miRNAs) are small non-coding RNAs (18–22 nt) that are crucial in regulating reproductive traits [3]. However, to date, no studies have been conducted on miRNAs related to reproduction in abalone. Thus, herein, we aimed to recognize miRNAs and miRNA–gene interactions in the ovaries and testes of abalones through an integrated analysis of miRNA and mRNA transcriptomes. This analysis elucidates the mechanistic role underlying reproduction and identifies possible molecular markers associated with shellfish reproductive processes, including sexual maturity and sex determination.

In this study, both mRNA and miRNA transcriptomes of the ovary and testis were sequenced from six sexually mature *H. discus hannai*. A total of 685 DEGs were found between both groups. These top 50 upregulated DEMs included *Verl*, *lev-9*, *Zp-like*, etc. *Verl* is associated with a species-specific mechanism to promote fertilization in the marine mollusk abalone [27]. *Zp-like* is expressed in the extracellular matrix that surrounds all mammalian oocytes, eggs, and early embryos. It is essential for oogenesis, fertilization, and preimplantation development [28]. These genes are highly expressed in the female *H. discus hannai* gonad and rarely or minimally expressed in the male *H. discus hannai* gonad. Among these downregulated DEGs, the top 50 genes included *Sox-30*, *Tssk*, *Tepp*, *Sperm-lysin*, etc. *Tssk* critically modulates reproductive cell differentiation and sperm activity [29]. *Tepp* displayed differential expression in sperm across various seasons [30]. Abalone *sperm-lysin*, a rapidly evolving reproductive protein, is a 16-kDa protein with a high positive charge. It plays a key role in dissolving the *VE* surrounding abalone oocytes [27]. These genes are highly expressed in the male *H. discus hannai* gonad and rarely or minimally expressed in the female *H. discus hannai* gonad. These findings depicted that these genes may be used as molecular markers to distinguish between male and female *H. discus hannai*.

Next, the biological functions of these DEGs were explored to determine their potential relevance in regulating reproductive traits. The analysis revealed that these DEGs were significantly correlated with various biological processes, including carbohydrate metabolic processes, starch and sucrose metabolism, glycosphingolipid biosynthesis, glycosaminoglycan degradation, amino and nucleotide sugar metabolism, etc. Importantly, energy metabolism plays a critical role in ATP production, which is essential for gonadal development, spermatogenesis, and oogenesis [24,31]. In this study, *Endoglucanase A, Fut1, XynX, Chit1, xyl3A, Alpha-amylase, celF*, and *Man2b1* were differently expressed between both groups of abalones, revealing that these genes are highly promising candidates for regulating oogenesis, spermatogenesis, and gonad development via energy metabolism. Further verification of the function of these genes in both genders of abalone will be undertaken in the future.

Multiple classes of sRNAs, such as miRNAs, regulate the processes of sex identification and differentiation, gonadal differentiation, and maturation [32,33]. Mature sequences of miRNAs are approximately 20–25 nt in length and participate in post-transcriptional gene repression [34]. The association between miRNA and RNA is complex, since one miRNA can potentially affect several genes, and one gene can potentially be affected by several miRNAs [35]. The investigation of associations between miRNA profiles and established target mRNAs, as well as the identification of possible correlation between novel miRNA and mRNA interactions, can be simplified by paired expression profiling of mRNA and miRNA [36,37]. The function of miRNAs and the identification of the miRNA-mRNA pairs involved in physiology could be understood through the profile analysis of combined miRNA and corresponding target mRNA [38]. Several key miRNAs, namely, let-7, miR-8, miR-100, miR-8*, miR-9*, and miR-315, have been implicated in sex identification, reproduction, differentiation, and growth. Remarkably, miR-8 and miR-8* may synergistically modulate the fruitless gene during female sexual differentiation and gonadal development in *Scylla paramamosain* [39]. To date, there have been no reports of integrated miRNA and target mRNA analyses in studies of sexual maturity and sex determination in *H. discus hannai*. The current study detected 57 DEMs between the DD_ovary_group and DD_testis_group abalones, of which there were 73 pairs (Table S7) with adversely associated miRNA-mRNA pairs, depicting that these specific miRNA-mRNA pairs participate in abalone reproduction traits. For example, novel_90 targeted *Verl*, novel_32 targeted *Cemip2*, novel_120 targeted *Tssk1B*, and so on. These miRNAs potentially target these genes to modulate reproduction, as they were reported to be associated with reproduction. Further, the enrichment assessment of these target DEGs demonstrated that these miRNA-mRNA pairs regulate the sexual maturity and sex determination of *H. discus hannai* by regulating carbohydrate metabolic processes, protein phosphorylation, kinase activity, and starch and sucrose metabolisms. However, additional research is necessary to determine the functional significance of the miRNA-mRNA pairs that were identified in the ovary and testis of *H. discus hannai*.

Finally, qRT-PCR analysis verified the accuracy of transcriptome sequencing. The findings showed that the relative candidate mRNA and miRNA expression trends strongly corroborated with the transcriptomic analysis, illustrating the reliability of the transcriptomic profile. Previous studies [40,41] have used FISH technology to examine the expression patterns of genes in abalone. Herein, FISH was also used to evaluate the gene expression localization within the gonads of *H. discus hannai*. The findings depicted that *Verl* was highly present in the ovaries of mature *H. discus hannai*, with weak positive signals observed in the ovaries of immature abalones. Similarly, *Tssk1B* depicted a high level in the testes of mature *H. discus hannai*, while weak positive signals were detected in the testes of immature abalones. These findings suggest that these genes, along with their role in sex determination, may serve as markers for assessing the sexual maturity of *H. discus hannai.*

## 4. Materials and Methods

### 4.1. Abalones and RNA Preparation

Sexually mature *H. discus hannai* (body weight 108.2 ± 9.7 g; 36 months old; three males and three females) with a visual gonad index (VGI) of 3 [42] were procured from Fuda Aquaculture (Jinjiang, Fujian Province, China). The VGI consists of four categories (0–3) that relate to changes in the size and shape of the gonad, wherein VGI 3 refers to abalones with a swollen gonad with rounded tip and are classified as sexually mature. Gonadal tissues were collected from three individuals of each sex, with each sample processed independently. To ensure sample integrity, the outer membrane of the gonads was carefully excised without disturbing the underlying digestive gland. Approximately 500 mg of gonadal tissue (eggs or sperm) was snap-frozen in liquid nitrogen and kept at −80 °C for storage. Total RNA was isolated from the gonadal samples using TRIzol reagent (Invitrogen, Waltham, MA, USA). The isolated RNA purity and integrity were then evaluated to ensure suitability for downstream analyses.

### 4.2. Transcriptome Sequencing and Data Processing

Approximately 3 µg of RNA per sample was processed to synthesize cDNA libraries. Index-coded samples underwent clustering via the TruSeq PE Cluster Kit v3-cBot-HS and the cBot Cluster Generation System (Illumina, San Diego, CA, USA). Sequencing employed the Illumina HiSeqX platform and produced 150 bp paired-end reads. Raw sequencing data underwent quality control using custom in-house Perl scripts, which eliminated reduced-quality reads, adapter sequences, and poly-N-harboring sequences to produce clean reads. Quality metrics, namely, Q20, Q30, and GC levels, were computed for the processed data.

Clean reads underwent alignment to the reference genome of *H. discus hannai* using TopHat v2.0.9 [43] under default parameters. The reference genome and gene model annotation files were acquired from Dr. Weiwei You (Xiamen University, Xiamen, China). Following assembly of mapped reads via Scripture (beta2) [44] and Cufflinks v2.1.1 [45,46], gene profiles were computed as fragments per kilobase (kb) of transcript per million mapped reads (FPKM) with Cuffdiff v2.1.1 [47], and gene counts were analyzed with HTSeq [48]. Furthermore, DEGs identification utilized the edgeR package [49], with selection criteria as follows: corrected *p*-value < 0.05 and |log_2_ fold change| ≥ 10. To interpret DEG functions, enrichment analyses were conducted using the Kyoto Encyclopedia of Genes and Genomes (KEGG) networks [50] and Gene Ontology (GO) [51], namely, molecular functions (MF), biological processes (BP), and cellular components (CC). These analyses were carried out via the database for Annotation, Visualization, and Integrated Discovery (DAVID) [52], with GO terms and networks considered significant at *p* < 0.05. Further, protein–protein interaction (PPI) axes were developed via STRING v10 [53].

### 4.3. sRNA Sequencing and Analysis

In all, 3 µg of total RNA/sample was employed to prepare the small RNA library. Following the manufacturer’s recommendations, single-end sequencing (50 bp) was performed on an Illumina HiSeq2500 at Novogene (Tianjin, China). Post-sequencing, clean reads were generated by eliminating sequences with poly-N, poly-A/T/G/C, adapter contamination, and reduced-quality reads from the raw information. The Q20, Q30, and GC levels of the raw data were computed to assess data quality. Next, we conducted downstream analyses on clean reads selected within a specified length range [54].

Small RNA tags underwent mapping to a reference sequence using Bowtie 1.0.1 [55], prior to search against the known miRNA database miRBase 20.0. To elucidate potential miRNAs, the modified software mirdeep2 [56] and srna-tools-cli (http://srna-tools.cm p.uea.ac.uk/ (accessed on 13 February 2025)) were employed, enabling secondary structure prediction. Novel miRNAs were predicted using an integrated approach combining miREvo 1.1 [57] and mirdeep2 [56] software. Differential expression between both groups was evaluated via the DESeq R package (1.8.3) [58] with *p*-values corrected via the Benjamini–Hochberg method [59]. An adjusted *p* < 0.05 threshold was considered significant for identifying DE.

### 4.4. miRNA Target Prediction and Functional Analyses

The miRNA’s target gene was identified via psRobot_v1.2 in miRanda-3.3a [60]. The roles of the estimated target genes were annotated using GOSeq [61] and KOBAS 2.0 [62] software. Further, Cytoscape (http://www.cytoscape.org) was employed for construction of the interaction networks between DE-miRNAs and their respective target genes.

### 4.5. Quantitative Real-Time PCR

We conducted gene expression assessment via qRT-PCR to further confirm the selected gene and miRNA expressions. Gene-specific primers were designed for the target genes, with *β-actin* as the internal control (Table 4). Specific and universal reverse primers were employed for miRNAs, with U6 employed as the reference gene for normalization [63]. Next, qRT-PCR assays were conducted using a CFX96 Real-Time System (Bio-Rad, Hercules, CA, USA). Amplification reactions (25 μL) comprised 12.5 μL of 2× M5 HiPer SYBR Premix EsTaq (withTli RNaseH), 0.5 μL of each forward and reverse primer (10 μM), 2 μL of cDNA (diluted 1:100), and 9.5 μL of nuclease-free water. The reaction conditions were as follows: a 30 s denaturation at 95 °C, with subsequent 40 cycles of 5 s denaturation at 95 °C, then a 30 s annealing/extension at 60 °C. Fluorescence intensities were recorded after each cycle. Relative target gene and miRNA expressions were estimated via the 2^−∆∆CT^ formula [64]. All experiments were repeated in triplicate, with three independent biological replicates.

### 4.6. Fluorescence In Situ Hybridization (FISH)

Fluorescein-labeled specific oligonucleotides were used as probes to target specific binding sequences. These probes hybridized with their complementary sequences, allowing for the fluorescent labeling of the target gene–probe hybrids. This enabled both the localization and quantification of the hybrids. Based on the *Verl* and *Tssk1B* gene sequence characteristics, FISH probes were designed, with their sequences detailed in Table 5. These probes were synthesized by Wuhan Servicebio Technology Co., Ltd. (Wuhan, China).

Gonadal tissues (ovary and testis) of *H. discus hannai* were fixed in an in situ hybridization fixative for >12 h and kept at 4 °C. These fixed tissues were sectioned into 3 mm thick blocks, dehydrated in an ethanol gradient, then cleared in xylene, prior to paraffin embedding. Sections were sliced to a 4 μm thickness, spread, and dried in a 62 °C oven for 2 h. The sections were immersed in dewaxing transparent liquid I for 15 min, followed by dewaxing transparent liquid II for 15 min, anhydrous ethanol I for 5 min, anhydrous ethanol II for 5 min, 85% alcohol for 5 min, 75% alcohol for 5 min, and DEPC water. Depending on the tissue type, the length of the fixed time, and the positioning of the index, different repair fluids were selected for repair, and the specific repair conditions are shown in Table 5. After natural cooling, the stroke circle was organized. Tissue-specific antigen retrieval was conducted as detailed in Table 5, and sections were digested with proteinase K (20 μg/mL) at 40 °C for different durations, also specified in Table 5. Following rinsing with DEPC water and PBS (thrice, 5 min each), pre-hybridization solution was introduced (dropwise) prior to a 1 h incubation at 40 °C. Subsequently, the solution was replaced with a probe-containing hybridization solution and allowed to react overnight in the incubator. All hybridization conditions are shown in Table 5. Post-hybridization washes included 10 min 2× SSC at 40 °C, 5 min 1× SSC at 40 °C, and a 10 min 0.5× SSC at room temperature. Sections can further be washed with formamide when nonspecific hybridization can be observed. Finally, an 8 min DAPI staining was performed without light, and an anti-fluorescence quenching sealing agent was added after washing.

Tissue sections were examined with a Nikon upright fluorescence microscope, and all images were captured for analysis. The ultraviolet (UV) excitation wavelength (λ) ranged from 330 to 380 nm with an emission λ of 420 nm, producing blue fluorescence. The green fluorescence emitted by FAM (488) was observed under an excitation λ of 465–495 nm and an emission λ of 515–555 nm, while CY3 red fluorescence was visualized with an excitation λ of 510–560 nm and an emission λ of 590 nm. Under UV excitation, nuclei stained with DAPI emitted blue fluorescence, and positive expressions were identified based on the respective fluorescein-labeled fluorescence. FAM (488) emitted green light, whereas CY3 produced red light, with the fluorescence intensity indicating the level of expression.

### 4.7. Statistics

All of the qRT-PCR data were expressed as mean ± standard deviation (SD). Statistical significance was evaluated using SPSS 19.0 (IBM Corp., Armonk, NY, USA). Significant differences were analyzed by two-tailed *t*-tests, with *p* < 0.05 considered statistically significant.

## 5. Conclusions

This study demonstrated the transcript and miRNA expressions of abalone gonads from DD_ovary_group and DD_testis_group abalones using HTS. This study revealed the identity of 137 miRNAs associated with the ovary and testis of *H. discus hannai* for the first time. Several key candidate mRNAs have detected and outlined a crucial functional miRNA–mRNA network related to abalone sexual maturity and sex determination. These key mRNAs and miRNAs may regulate sexual maturation and sex determination of abalone via carbohydrate metabolic processes, phosphotransferase activity, and starch and sucrose metabolisms. Multiple mRNAs and miRNAs were also selected for further analysis, and the expression was analyzed using RT-qPCR and FISH analyses. Overall, these findings enrich the genetic information resources of abalone, suggest a new direction of the mechanistic role of reproductive traits of shellfish, and offer a theoretical basis for improving the reproductive efficiency of abalone.

## Figures and Tables

**Table 1:** The results of RNA quality.

Sample Name	Raw Reads	Raw Bases	Clean Reads	Clean Bases	Error Rate	Q20 (%)	Q30 (%)	GC pct (%)
DD_ovary_1	44,600,466	6.69G	43,341,220	6.5G	0.03	94.87	87.52	45.16
DD_ovary_2	47,513,192	7.13G	46,428,612	6.96G	0.03	95.07	87.84	45.29
DD_ovary_3	44,400,154	6.66G	43,435,352	6.52G	0.03	94.53	86.61	43.46
DD_testis_1	41,592,936	6.24G	40,138,628	6.02G	0.03	97.25	92.19	43.31
DD_testis_2	45,197,534	6.78G	44,052,766	6.61G	0.03	97.06	91.82	43.38
DD_testis_3	44,737,000	6.71G	42,770,608	6.42G	0.03	96.86	91.44	43.87

**Table 2:** Small RNA sequencing data.

Sample	Total_Reads	N% > 10%	Low Quality	5_adapter_ contamine	3_adapter_null or insert_null	With ployA/T/G/C	Clean Reads
DD_ovary_1	12,604,189 (100.00%)	1 (0.00%)	0 (0.00%)	2907 (0.02%)	263,476 (2.09%)	3627 (0.03%)	12,334,178 (97.86%)
DD_ovary_2	11,763,492 (100.00%)	319 (0.00%)	0 (0.00%)	11,695 (0.10%)	91,539 (0.78%)	35,964 (0.31%)	11,623,975 (98.81%)
DD_ovary_3	11,148,631 (100.00%)	270 (0.00%)	0 (0.00%)	11,784 (0.11%)	134,483 (1.21%)	30,690 (0.28%)	10,971,404 (98.41%)
DD_testis_1	11,558,139 (100.00%)	0 (0.00%)	0 (0.00%)	2243 (0.02%)	359,076 (3.11%)	2194 (0.02%)	11,194,626 (96.85%)
DD_testis_2	11,598,714 (100.00%)	0 (0.00%)	0 (0.00%)	7447 (0.06%)	247,111 (2.13%)	8378 (0.07%)	11,335,778 (97.73%)
DD_testis_3	11,563,092 (100.00%)	0 (0.00%)	0 (0.00%)	2606 (0.02%)	580,785 (5.02%)	2072 (0.02%)	10,977,629 (94.94%)

**Table 3:** Differentially expressed miRNAs (DEMs) and their DE target mRNAs (target DEGs).

Target DEG id	Target DEG Name	log_2_Fold Change	padj	Related miRNA
HDH_G17212	*Alpha-amylase*	11.5793044	8.34E-14	hdh-miR-133-3p
HDH_G10591	*ATP-binding cassette sub-family C member 3* (*Abcc3*)	10.69052989	3.88E-14	novel_32
HDH_G16571	*Chitin binding Peritrophin-A domain* (*CbpA*)	10.02494546	1.08E-09	hdh-miR-1990
HDH_G27689	*Chitotriosidase-1* (*Chit1*)	12.04039088	1.27E-14	hdh-miR-2722
HDH_G29537	*Endoglucanase F* (*CelF*)	13.61183447	2.04E-18	novel_19
HDH_G17091	*Exoglucanase XynX* (*XynX*)	10.07419645	2.56E-08	novel_3
HDH_G14591	*Inactive cell surface hyaluronidase CEMIP2* (*Cemip2*)	14.81063531	3.02E-25	novel_32
HDH_G05585	*Lambda-crystallin* (*Cryl1*)	10.20412277	1.31E-09	hdh-miR-2722, novel_19
HDH_G13593	*Lectin C-type domain* (*Ctls*)	10.28631776	2.36E-09	novel_90
HDH_G08135	*Low-density lipoprotein receptor domain class A* (*LdlrA)*	14.26186176	5.70E-19	novel_90
HDH_G26485	*Lysosomal alpha-mannosidase* (*Man2b1)*	12.22683725	4.35E-17	hdh-miR-92
HDH_G25248	*Mannan endo-1,4-beta-mannosidase* (*ManA*)	12.05066365	2.40E-14	hdh-miR-2722
HDH_G06159	*Phenolphthiocerol/phthiocerol polyketide synthase subunit B* (*PpsB*)	11.98933126	4.70E-19	novel_18
HDH_G14448	*Prestin* (*Slc26a5*)	10.15083509	1.76E-12	hdh-miR-2722
HDH_G06399	*Protein lev-9* (*lev-9*)	18.16934184	1.96E-35	novel_101
HDH_G15576	*Protein unc-93 homolog A* (*Unc93A*)	10.02044907	1.97E-11	novel_3
HDH_G15755	*Ras-related protein Rab-3A* (*Rab3A*)	10.03697922	1.02E-12	novel_90
HDH_G27130	*Saposin-like type B, region 1* (*SapB1*)	12.75468774	2.94E-15	novel_32
HDH_G25000	*Sodium- and chloride-dependent glycine transporter 2* (*Slc6a5*)	11.09950664	6.36E-15	novel_18
HDH_G21292	*Slc5a6*	10.49241993	1.19E-13	novel_18
HDH_G21270	*Scl52a3B*	10.96700804	8.10E-15	novel_53
HDH_G16512	*Sulfotransferase 1A1* (*Sult1A1*)	10.29513478	3.53E-10	novel_18
HDH_G29406	*Transcription factor Hes4A* (*Hes4A*)	11.25346065	2.90E-16	novel_42
HDH_G24939	*Transforming growth factor-beta-induced protein ig-h3* (*Tgfbi*)	11.38049792	2.95E-13	novel_32
HDH_G12983	*Vitelline envelope sperm lysin receptor* (*Verl*)	15.48186129	1.00E-33	novel_90
HDH_G17385	*Verl*	19.59400277	8.33E-54	novel_53
HDH_G15719	*Verl*	18.78354843	9.64E-49	novel_1, novel_8
HDH_G14936	*Xanthine dehydrogenase* (*Xdh*)	10.06294149	1.01E-09	novel_53
HDH_G06239	*Xylan 1,4-beta-xylosidase* (*Xyl3A*)	11.28666914	2.85E-11	novel_19, novel_8
HDH_G31217	*Ankyrin repeats* (*AR*)	−13.11878319	9.27E-25	hdh-miR-1994a, hdh-miR-1994b
HDH_G04117	*E3 ubiquitin-protein ligase Dcst1* (*Dcst1*)	−10.29220265	5.34E-15	novel_57
HDH_G27469	*Inactive serine/threonine-protein kinase Tex14* (*Tex14*)	−12.05306941	3.31E-137	novel_120
HDH_G02229	*Leucine rich repeat* (*LRR*)	−13.50459197	1.33E-26	novel_120
HDH_G08545	*RING-variant domain*	−14.2614608	5.53E-29	novel_6
HDH_G05810	*Stabilizer of axonemal microtubules 4* (*Saxo4*)	−10.25719851	4.38E-55	hdh-miR-31
HDH_G26330	*Testis-specific serine/threonine-protein kinase 1* (*Tssk1B*)	−10.66140224	1.63E-58	novel_120, novel_6
HDH_G12498	*Transcription factor Sox-30* (*Sox-30*)	−13.81154742	5.52E-27	novel_120

**Table 4:** Pairs of primer sequences.

Primer	Sequence (5′-3′)
*Saxo4*-qF	GTTCAAGGGTCTTCGAGGCA
*Saxo4*-qR	CGTGAAATAACCGGGCTGCT
*Tssk1B*-qF	CCACCATTCTGACCATCCCT
*Tssk1B*-qR	CCTCCTTCTTCTTCCTCTCGG
*AR*-qF	GAAAATGGGATCCTCGGCTG
*AR*-qR	TTACCCCTCACCGCTTGAAT
*Verl*-qF	GACTTCCGGGCCATCTGTAA
*Verl*-qR	ACGTTGGAGTTCTGTCTCCT
*Man2b1*-qF	GGAGGCTAAAGCGCTCATCA
*Man2b1*-qR	CCGAACTTTTGCTCAGCGTT
*β-actin*-qF	GGTATCCTCACCCTCAAGT
*β-actin*-qR	GGGTCATCTTTTCACGGTTG
hdh-miR-92	AATTGCACTTGTCCCGGCCTGC
novel_1	AATTGCACTCGTCCCGGCCTGCAA
hdh-miR-1994b	TGAGACAGTGTGTCCTCCCTCA
hdh-miR-31	AGGCAAGATGTTGGCATAGCT
novel_6	TCGAGGAAGTAGAAGACCTTGACGT

**Table 5:** Probe information and repair conditions.

Probe Name	Digestive Condition	Probe Sequence (5′-3′)	Repair Condition	Probe Concentration	Hybridization Temperature	Name of the Corresponding Signal Probe
*Verl*	Proteinase K was digested at 40 °C for 10 min.	TATGCAGGTAATGGTGCCGTCAAG /GTAATCGACCCTTCCGGTTCCAAG /TTGCGAATCTTGTGTTCGTCCTCG	Tissue sections were kept in a citric acid solution (pH 6.0) repair box and incubated in a water bath at 90 °C for 48 min.	500 nM	40 °C	CY3
*Tssk1B*	Proteinase K was digested at 40 °C for 10 min.	CTCGTGAAGGACCAGCAGAGAAGC /CATAGGACCCACAGAAGGTCTCCATC /GTTCTCGGCAGTCTTTAGACACCTGC	Tissue sections were kept in a citric acid solution (pH 6.0) repair box and incubated in a water bath at 90 °C for 48 min.	500 nM	40 °C	FAM (488)

## Data Availability

The data presented in this study are available upon request from the corresponding authors.

## Supplementary Materials

The following supporting information can be downloaded at: https://www.mdpi.com/article/10.3390/ijms26073235/s1.

## Abbreviations

The following abbreviations are used in this manuscript:

miRNA	microRNA
*H. discus hannai*	*Haliotis discus hannai*
DE	differentially expressed
*Tssk*	*Testis-specific serine/threonine-protein kinase*
*Verl*	*Vitelline envelope sperm lysin receptor*
FISH	fluorescence in situ hybridization
UTRs	untranslated regions
HTS	high-throughput sequencing
DEGs	differentially expressed genes
DEMs	differentially expressed miRNAs
*brat*	*Brain tumor protein*
*Ppn*	*Papilin*
*Fut2*	*Galactoside alpha-(1,2)-fucosyltransferase 2*
*Tuba1A*	*Tubulin alpha-1A chain*
*Fasn*	*Fatty acid synthase*
*Inmt*	*Indolethylamine N-methyltransferase*
*Tpo*	*Thyroid peroxidase*
*hFat3*	*Protocadherin Fat 3*
*Zp-like*	*Zona pellucida-like domain*
*SOX**-**30*	*Transcription factor SOX-30*
*Tepp*	*Testis, prostate* and *placenta-expressed protein*
*HSF1*	*Heat shock factor protein 1*
*Saxo4*	*Stabilizer of axonemal microtubules 4*
*AR*	*Ankyrin repeats*
*Man2b1*	*Lysosomal alpha-mannosidase*
KEGG	Kyoto Encyclopedia of Genes and Genomes
GO	Gene Ontology
MF	molecular functions
BP	biological processes
CC	cellular components
PPI	protein–protein interaction
qRT-PCR	Quantitative real-time PCR
SD	standard deviation
*Abcc3*	*ATP-binding cassette sub-family C member 3*
*CbpA*	*Chitin binding Peritrophin-A domain*
*Chit1*	*Chitotriosidase-1*
*CelF*	*Endoglucanase F*
*XynX*	*Exoglucanase XynX*
*Cemip2*	*Inactive cell surface hyaluronidase CEMIP2*
*Cryl1*	*Lambda-crystallin*
*Ctls*	*Lectin C-type domain*
*LdlrA*	*Low-density lipoprotein receptor domain class A*
*ManA*	*Mannan endo-1,4-beta-mannosidase*
*PpsB*	*Phenolphthiocerol/phthiocerol polyketide synthase subunit B*
*Slc26a5*	*Prestin*
*lev-9*	*Protein lev-9*
*Unc93A*	*Protein unc-93 homolog A*
*Rab3A*	*Ras-related protein Rab-3A*
*SapB1*	*Saposin-like type B, region 1*
*Slc6a5*	*Sodium- and chloride-dependent glycine transporter 2*
*Sult1A1*	*Sulfotransferase 1A1*
*Hes4A*	*Transcription factor Hes4A*
*Tgfbi*	*Transforming growth factor-beta-induced protein ig-h3*
*Xdh*	*Xanthine dehydrogenase*
*Xyl3A*	*Xylan 1,4-beta-xylosidase*
*Dcst1*	*E3 ubiquitin-protein ligase Dcst1*
*Tex14*	*Inactive serine/threonine-protein kinase Tex14*
*LRR*	*Leucine rich repeat*
